# Integrative benchmarking and automation of clonal reconstruction of somatic mutations in single-sample tumor genome analysis

**DOI:** 10.1186/s12859-026-06589-8

**Published:** 2026-08-05

**Authors:** Marina Masliakova, Steve Lefever, Jo Vandesompele

**Affiliations:** 1 pxlence bv, Building RTP (entrance 97), Corneel Heymanslaan 10, Ghent, 9000 Belgium; 2 Department of Biomolecular Medicine, Ghent University, Building the Core (entrance 37), Corneel Heymanslaan 10, 9000 Ghent, Belgium

**Keywords:** Clonal reconstruction, Tumor evolution, Cancer genomics, CtDNA, Liquid biopsy

## Abstract

**Background:**

Accurate identification of truncal mutations, those present in all tumor cells, is essential for understanding tumor evolution and guiding downstream analyses in cancer genomics, including detection of minimal residual disease. Existing clonal reconstruction tools vary in accuracy, specificity, and computational efficiency, and, to our knowledge, no standardized workflow exists for systematic truncal mutation extraction.

**Results:**

We benchmarked five clonal reconstruction tools on simulated tumor genomes from the DREAM challenge using true positive rate, false positive rate, and runtime. Based on the results, we developed TruncalFlow, a containerized pipeline for automated clonal reconstruction and truncal mutation extraction. Applied to real tumor genomes from 30 cancer types available in TCGA, it enabled analyses of mutation types, recurrence among tumor types, and cancer driver annotation, identifying biologically relevant truncal mutations enriched for driver events. TruncalFlow provides a scalable, accurate framework for systematic truncal mutation analysis in cancer genomes.

**Conclusions:**

TruncalFlow enables robust and automated identification of truncal mutations from tumor sequencing data, supporting accurate clonality assessment across cancer types. By facilitating scalable extraction of both driver and non-driver truncal variants, it provides a practical framework linking tumor evolution analysis to translational and liquid biopsy applications.

**Supplementary Information:**

The online version contains supplementary material available at https://doi.org/10.1186/s12859-026-06589-8.

## Background

Genome-wide analyses of tumor genomes have profoundly transformed our understanding of cancer and enabled the development of precision oncology approaches. Despite this progress, it remains challenging to detect relapse or recurrence early. Novel strategies are needed to improve the monitoring of therapeutic response, detect resistance, and guide interventions tailored to individual tumor profiles.

**Fig. 1:**
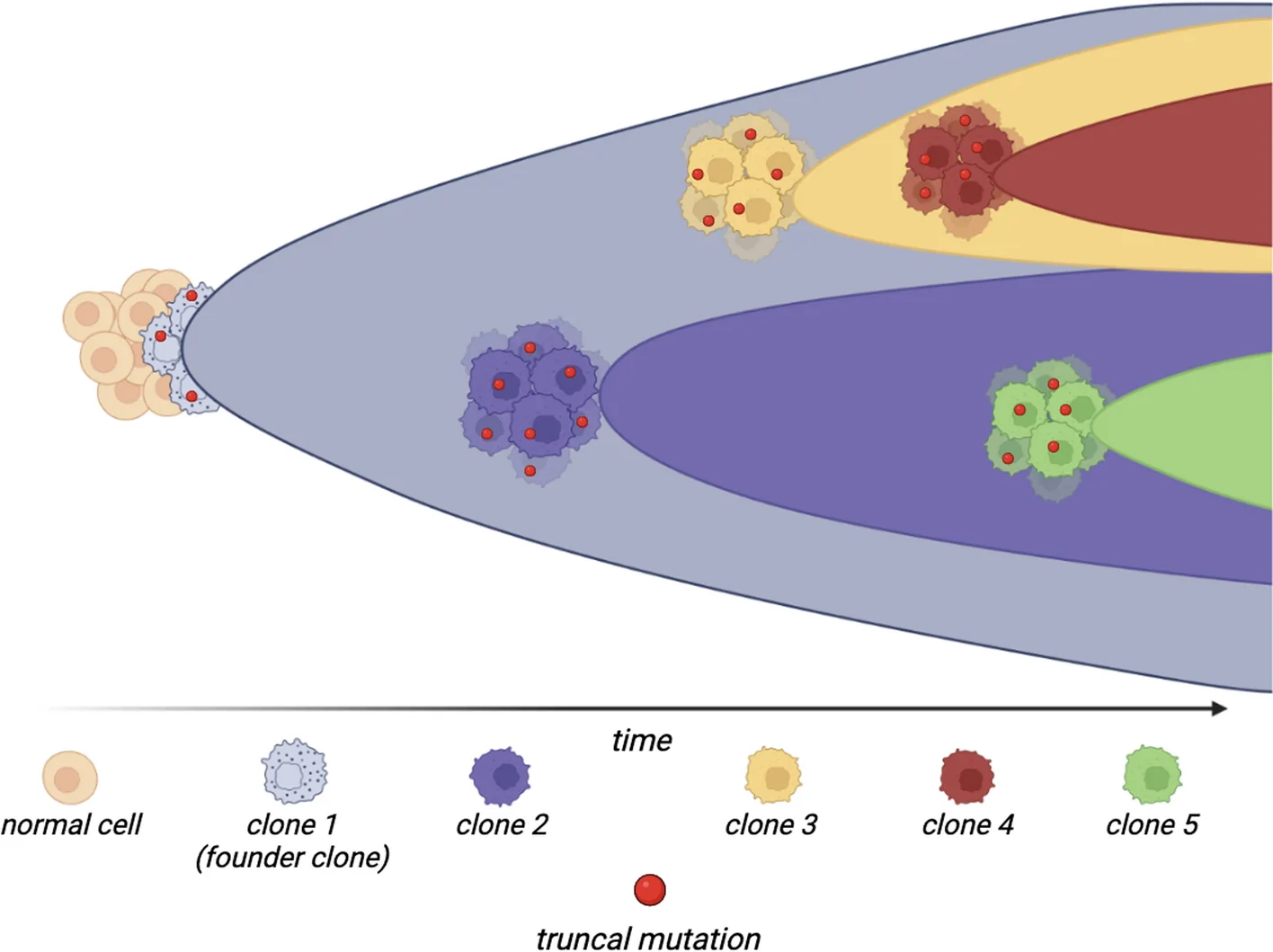
Clonal evolution of mutations in cancer tissue. Mutations that are present in all clones, including the founder clone, are truncal

Fundamentally, cancer genomics has revealed that tumors do not arise from the alteration of a single gene, but rather through a complex, patient-specific evolutionary process. Tumor cells acquire somatic mutations over time, resulting in the emergence of genetically distinct subpopulations, or clones [1]. Each clone reflects a phase of tumor evolution, and the mutations shared by all cancer cells − known as truncal or founder mutations − represent the earliest events in tumorigenesis [2]. In contrast, subclonal or branch mutations arise later and contribute to tumor heterogeneity (Fig. 1). Understanding the clonal structure of tumors thus provides critical insight into their biology, therapeutic vulnerabilities, and resistance mechanisms.

Clonal heterogeneity poses a major challenge for both cancer treatment and disease monitoring. Mutations from different clones may confer differential drug sensitivity or resistance, and treatment targeting subtruncal mutations may fail to eliminate the bulk of the tumor. However, truncal mutations, present in all tumor cells, offer a more stable and representative target for therapy and for the detection of residual disease. Indeed, monitoring truncal mutations can provide a more comprehensive and robust view of tumor burden and therapeutic response [3]. Therefore, there is growing interest in bioinformatics methods that can reliably identify truncal mutations in tumor samples.

Computational tools for clonal deconvolution aim to reconstruct the clonal composition of a tumor and identify mutations associated with each clone. Early approaches focused on using either simple somatic mutations (SSMs) like single nucleotide variants (SNVs) and small insertions and deletions (indels), or copy number alterations (CNAs) for clonal reconstruction [4, 5]. The core of such reconstruction is to group mutations by their estimated prevalence across the tumor cell populations. Central to these methods is the concept of variant allele frequency (VAF), which serves as a proxy for the cellular fraction of each mutation [6, 7]. By clustering mutations with similar VAFs, these algorithms attempt to identify groups that represent distinct clones, with one cluster typically corresponding to truncal mutations. Over time, more sophisticated tools were developed that incorporate both SSMs and copy number variation (CNV) data to account for the influence of copy number changes on VAFs. Such clonal reconstruction tools are based on a mathematical model framework to translate observed VAFs into estimates of cellular prevalence or clonal composition, ideally correcting for CNVs. These frameworks can range from Bayesian hierarchical models [8, 9] to frequentist maximum likelihood models [10, 11] or combinatorial phylogenetic approaches [5, 12]. Some tools, such as PhyloWGS [13] and FastClone [11], integrate phylogenetic inference into their reconstruction to output both clonal assignments and the evolutionary relationships between clones.

Notably, while these tools provide important insights into tumor phylogeny, they often remain inaccessible to non-specialists. They typically require extensive data preprocessing, specific input formats, and detailed interpretation of complex output. In practice, extracting a definitive set of truncal mutations from these tools remains a cumbersome task, limiting their adoption for use in translational research or in the clinical setting. To address this gap, we present an integrative benchmarking study of representative clonal deconvolution tools and introduce **TruncalFlow** − a streamlined command-line wrapper designed to automate the identification of truncal mutations from tumor genomic data. To our knowledge, this is the first tool that delivers a full pipeline for extracting truncal mutations from single-sample input, implemented in a containerized format. Beyond accepting standard input mutation data, it is customizable, allowing users to specify parameters to filter for specific variant types (e.g., SNVs or indels), or functional categories (e.g., protein coding, missense), with an option to combine both filters. Our benchmarking and tool development are particularly relevant in the context of liquid biopsy technologies that provide a minimally invasive means of capturing tumor-derived genetic material, such as circulating tumor DNA (ctDNA). Liquid biopsies are extensively used for mutation profiling, monitoring therapy response, and detecting drug resistance [14]. Response monitoring benefits from prior single-tumor sample clonal analysis, since it bypasses the need for multi-region biopsies and supports dynamic tracking of truncal mutations over time. Consequently, our study focuses on single-sample clonal reconstruction methods, aligning both with emerging diagnostic practices and the practical needs of precision oncology [15–17].

## Implementation

### Datasets

Two datasets were used in this study: one with known ground truth for benchmarking, and another for using the selected optimal tool on empirical cancer genome sequencing data.

For benchmarking, the simulated dataset from the ICGC-TCGA DREAM Somatic Mutation Calling - Tumour Heterogeneity Challenge [18] was taken, which contains 51 tumor samples with predefined clonal structures, enabling objective evaluation of computational methods. Variant Calling Format (VCF) files with SNVs and Battenberg outputs with allele-specific CNVs served as inputs for clonal reconstruction. The ground-truth clonal structures for each sample were used to evaluate the accuracy of each method.

For experiments with TruncalFlow, based on QuantumClone, the tool that showed the most suitable trade-off for truncal mutation identification in our benchmarking, we used data from The Cancer Genome Atlas (TCGA) Research Network [19]. This dataset covers 30 tumor types, with VCF and CNV files analyzed for clonal reconstruction.

### Clonal reconstruction methods

Five clonal reconstruction tools were benchmarked in this study: SciClone (v1.1) [10], PhyloWGS (v1.0-rc2) [13], QuantumClone (v1.0.0.9) [20], PyClone-VI (v0.1.0) [21], and FastClone (1.0.9) [11]. Selection criteria for the tools included public availability, single-sample analysis, prominence in the field, and diversity in algorithmic features such as CNV integration and evolutionary tree reconstruction. Each method was run using default parameters in single-sample mode to determine the optimal tool for annotating mutations as truncal or subtruncal. Below, we outline how each tool performs clonal reconstruction on a single tumor sample and provide a comparative summary of its key features.

SciClone minimizes the impact of subclonal CNVs and loss-of-heterozygosity (LOH) when estimating tumor subclones. It uses VAF data together with CNV and LOH annotations to filter out variants in non–copy-number-neutral or LOH-affected regions. The remaining variants are clustered with a variational Bayesian mixture model, assuming each subclone follows a beta-distributed VAF profile. Cluster assignments are iteratively refined via variational Bayesian inference until convergence. Low-confidence clusters − fewer than three mutations or <0.5% of total variants − are pruned, and the tool outputs the final subclone assignments with their associated mutations.

PhyloWGS integrates SSMs and CNVs to reconstruct subclonal phylogenies. Building on the Bayesian PhyloSub model [22], it uses a Dirichlet process prior to mutation clustering without predefining the number of subclones and employs Markov chain Monte Carlo (MCMC) sampling to explore possible phylogenetic trees. CNVs are incorporated by converting them into “pseudo-SSMs” and adjusting allele frequencies to account for copy number state, tumor purity, and the evolutionary timing of CNVs relative to SSMs. The output includes ranked phylogenetic trees, mutation clusters, and estimated population frequencies for each subclone.

QuantumClone improves subclonal reconstruction by correcting variant allele frequencies for CNV effects and tumor purity. Using SNV and copy number data, it estimates the cellular prevalence for each mutation and clusters them with an expectation–maximization algorithm that accounts for uncertainty in chromosomal configuration. Mutations from low-copy-number regions are weighted more heavily, while amplified regions contribute less due to ambiguity. The optimal number of subclones is selected by the Bayesian information criterion, and the tool outputs subclones with their assigned mutations.

PyClone-VI extends the original PyClone model [8] with a variational inference framework for greater scalability and speed. Using somatic mutation and copy number data, it estimates cellular prevalences and clusters mutations with a finite mixture model, replacing the Dirichlet process approach of the original version. The posterior distribution is approximated via variational inference optimization rather than MCMC sampling, with cellular prevalences restricted to a discrete grid to efficiently precompute likelihoods. The result is a set of subclonal populations defined by shared cellular prevalences, suitable for downstream phylogenetic analysis.

FastClone reconstructs the subclonal architecture from the bulk DNA sequence of a single tumor sample, incorporating SNVs from all genomic regions, including CNV-affected loci. It estimates mutation prevalence via maximum likelihood and infers the number of subclones by identifying peaks in allele frequency distributions using Gaussian kernel density estimation. SNVs are assigned to subclones through a probabilistic framework that evaluates possible evolutionary scenarios, selecting the configuration with the clearest prevalence structure. The output includes the tumor phylogeny, subclone definitions, and associated mutations.

### Benchmarking process

For all tools, SNV and CNV data were obtained from VCF and Battenberg files, respectively. Each method was executed using its command-line or R package scripts. Analyses were run on the Ghent University high-performance computing cluster, with 16 nodes equipped with 2*× *64-core processors and 940 GB of RAM per node.

#### Data preprocessing

SciClone required VAFs for SNVs, CNV data with regional ploidy, and LOH annotations; LOH regions were extracted from Battenberg output as loci with a minor allele copy number of zero. PhyloWGS inputs were generated using its original parser: CNVs were processed with *parse_cnvs.py* to extract copy number states and cellular prevalence, then combined with SSM data via *create_phylowgs_inputs.py*. Two sets of data were prepared for QuantumClone − (1) SNV data with genotype information and (2) SNV data with CNV profiles to determine copy number–adjusted genotypes. PyClone-VI used a single dataframe containing SNV and CNV data. FastClone was run directly from VCF and Battenberg files without additional formatting.

#### Execution of clonal reconstruction tools

SciClone was run via the *sciClone* R function with VAFs, CNV profiles, and LOH annotations, excluding affected regions. PhyloWGS was executed using the *multievolve.py* script with four parallel MCMC chains to explore tumor phylogenetic trees and mutation clusters. QuantumClone was run in R with the *One_step_clustering* function, using two input formats − (1) SNVs with genotypes and (2) SNVs with CNV-adjusted genotypes − keeping other parameters default. PyClone-VI was executed with its *fit* function, using a beta-binomial model on SNV and CNV data, and FastClone was run via *load-mutect-battenberg* to assign SNVs to subclones and estimate subclone proportions.

#### Metrics

Tool accuracy was assessed using three metrics for truncal mutation classification: number of mutation clusters identified, TPR − the proportion of true truncal mutations correctly identified, and FPR − the proportion of non-truncal mutations incorrectly labeled as truncal. Ground truth labels served as the reference for the metrics.

Because the number of ground-truth truncal mutations varied across samples, weighted averages were used as performance metrics, scaling each sample’s contribution by its number of true truncal mutations:

$$\begin{aligned} \text {weighted TPR} = \frac{\sum \limits _{i=1}^{n} (TPR_i \cdot N_{\text {truncal}, i})}{\sum \limits _{i=1}^{n} N_{\text {truncal}, i}} \end{aligned}$$

$$\begin{aligned} \text {weighted FPR} = \frac{\sum \limits _{i=1}^{n} (FPR_i \cdot N_{\text {truncal}, i})}{\sum \limits _{i=1}^{n} N_{\text {truncal}, i}} \end{aligned}$$

where $TPR_i$ and $FPR_i$ are the true and false positive rates for sample *i*, and $N_{truncal, i}$ represents the number of truncal mutations in sample *i*, derived from the ground truth.

### TruncalFlow development

To facilitate the identification of truncal mutations, we developed TruncalFlow, a command-line workflow that integrates preprocessing, clonal reconstruction, and postprocessing steps into a fully automated pipeline. The tool was implemented in Python for input handling, filtering, and output generation, and in R for executing the QuantumClone clustering step. TruncalFlow accepts as input single or multiple VCF files, with optional CNV data in Battenberg or major/minor allele format; CNV integration is recommended as it improves clustering accuracy. The preprocessing options include basic quality filters and user-defined constraints on variant types or functional categories.

Following QuantumClone clustering, TruncalFlow identifies the truncal cluster by selecting the cluster ID with the maximum estimated cellular prevalence from the *centers.csv* output file by the QuantumClone clustering step, which represents the most ancestral clonal population. All mutations assigned to this cluster are then extracted from the *clustering.csv* and annotated with their mutation type (SNV or indel), parsed from the input VCF, to generate the final list of truncal mutations.

The workflow produces per-sample tables of truncal mutations annotated with genomic coordinates, alleles, mutation type, cluster assignment, and estimated cellular prevalence. It is designed for high-throughput use, supporting multiple samples in parallel, and is containerized using Singularity to ensure reproducibility and ease of deployment. In Supplementary Section, a workflow scheme is provided (Fig. S3).

TruncalFlow supports two execution modes via *–executor* flag: pbs (requiring a PBS scheduler on HPC clusters) or local. Resource needs scale with input variant count and sample number, and can be adjusted using *–cpus*, *–mem*, and *–time*. In practice, 1 CPU and 4–6 GB RAM are typically sufficient for up to about 10,000 variants per sample, while larger inputs (up to 80,000 variants) require scaling to 2–4 CPUs and up to 32 GB RAM. For datasets with hundreds of thousands of variants, additional computational resources should be allocated accordingly.

## Results

### Benchmarking

The DREAM Challenge benchmarked subclonal reconstruction algorithms but reported results only for the five top-performing tools, limiting comparative assessment. Several of these methods are not publicly available or lack documentation for reproducible use. To overcome these limitations and ensure compatibility with our pipeline, we performed an independent benchmarking including publicly available top-performing tools from the DREAM Challenge and additional widely used algorithms.

**Fig. 2:**
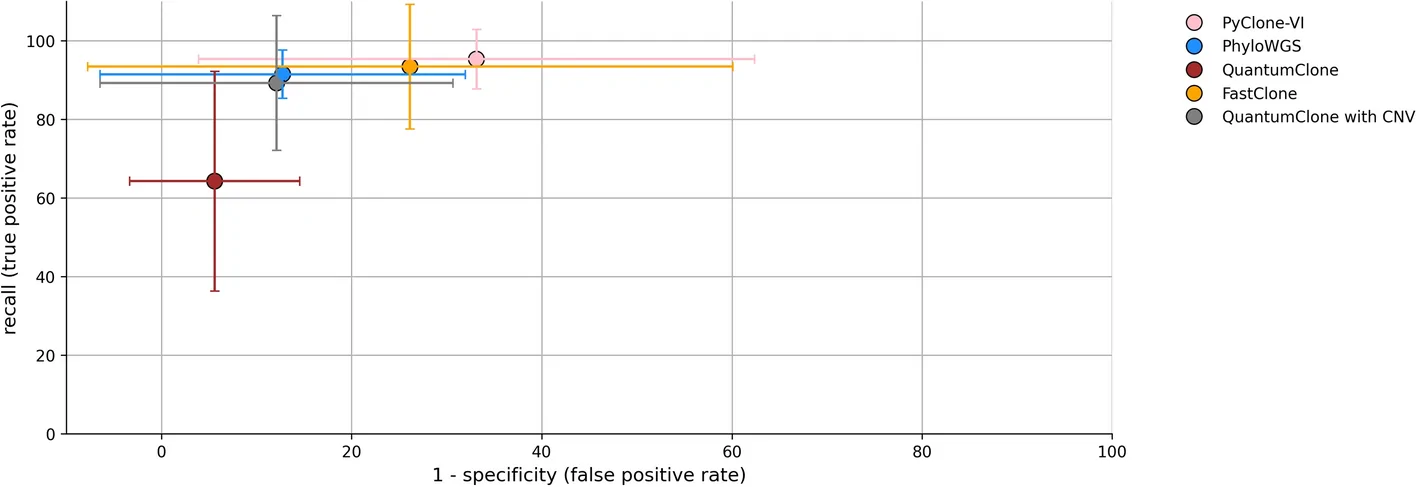
Comparison of weighted TPR and FPR across clonal reconstruction methods. Values are summarized over samples for each method, with error bars indicating the standard deviation across samples

The number of mutation clusters (clones) per sample revealed initial differences among the clonal reconstruction tools. SciClone consistently assigned all variants to a single cluster, failing to delineate subclonal clusters. QuantumClone (with and without CNV input) and FastClone determined the correct number of clusters in more than 75% of the samples, whereas PyClone-VI and PhyloWGS displayed lower accuracy and less consistent results in cluster numbers. Next, we compared the performance of the methods in terms of truncal mutation identification. PyClone-VI achieved the highest sensitivity in assigning mutations to correct clusters, but at the expense of a markedly elevated false positive rate (FPR), reflecting frequent misclassification of subtruncal mutations as truncal. QuantumClone without CNV correction showed the lowest FPR, but this conservative approach came with reduced sensitivity. Incorporating CNV data improved the true positive rate (TPR) metric for QuantumClone while maintaining a low FPR, outperforming PyClone-VI’s excessive FPR and resulting in the most balanced performance profile, which is illustrated in Fig. 2.

Additional performance metrics, including runtime and memory requirements, further differentiated the tools. FastClone was the most computationally efficient but tended to merge subclonal clusters, resulting in lower granularity of reconstruction, while PhyloWGS provided detailed reconstructions, including tumor phylogeny (Table 1), but was limited by excessive resource demands (Table S1). QuantumClone completed the analyses with moderate resource requirements, demonstrating both scalability and robustness.

In certain instances, tool performance varied by tumor type, likely driven by underlying genomic characteristics rather than purity or ploidy alone. FastClone outperformed both PhyloWGS and QuantumClone with CNV (higher TPR and lower FPR) in all three kidney chromophobe (KICH) samples and both liver hepatocellular carcinoma (LIHC) samples. In contrast, PhyloWGS outperformed both QuantumClone with CNV and FastClone in three cases of multiple myeloma (MM). Additionally, we examined whether QuantumClone with CNV systematically missed specific ground truth truncal mutations across samples. We found that mutations missed by QuantumClone were single-sample-specific events not reproduced across other samples within the same tumor type or different tumor types. This indicates that QuantumClone’s performance limitations are not due to systematic failure to detect specific mutation types, but rather sample-specific challenges.

These results underscore that QuantumClone with CNV integration provides a robust trade-off between accuracy and efficiency. However, it does not attempt to reconstruct phylogenetic relationships between clones, as the authors note that correctly inferring tumor phylogeny remains challenging [20]. Since the identification of truncal mutations does not require tree reconstruction, QuantumClone with CNV correction was selected as the core clonal reconstruction algorithm for TruncalFlow, as our objective was to identify a high-confidence set of truncal mutations. In this setting, minimizing false truncal calls is more important than maximizing sensitivity, since a false truncal assignment can mislead downstream biological interpretation and assay design.

**Table 1:** Comparison of features among clonal reconstruction methods

Method	CNV	Cellular prevalence	Clonal prevalence	Multi-sample option	Phylogeny output
SciClone	Yes	No	No	Yes	No
PhyloWGS	Yes	Yes	No	Yes	Yes
QuantumClone	Yes	Yes	No	Yes	No
PyClone-VI	Yes	Yes	No	Yes	No
FastClone	Yes	No	Yes	Yes	Yes

Proportion of malignant cells with the mutation in the sample. Proportion of malignant cells in the sample that belong to each inferred clone or subclone. QuantumClone can also be run without CNV information

### Downstream applications of TruncalFlow

To demonstrate the utility of TruncalFlow in real-life clinical samples, it was applied to 1005 TCGA tumor samples to investigate the properties of truncal mutations across 30 different cancer types.

#### Mutation type distribution in truncal variants

We compared the distribution of SNVs and indels in the complete variant set with the subset of truncal mutations identified by TruncalFlow. Across tumor types, SNVs dominated both datasets (i.e. total and truncal mutations) in line with previous reports [23, 24]. The relative proportions of mutation types remained largely consistent, indicating that clonal inference did not introduce a systematic bias toward SNVs or indels (Fig. S1).

Distinct tumor-type–specific patterns were observed. COAD and READ showed the highest fraction of indels, which increased from approximately 20% in the original VCFs to almost 30% among truncal mutations (Table S2). In contrast, BLCA, LUAD, LUSC, and SKCM consistently exhibited the lowest indel fractions (<10%) among all or clonal mutations. Notably, GBM displayed a distinctive enrichment of indels among recurrent truncal events.

#### Recurrence of truncal mutations

**Fig. 3:**
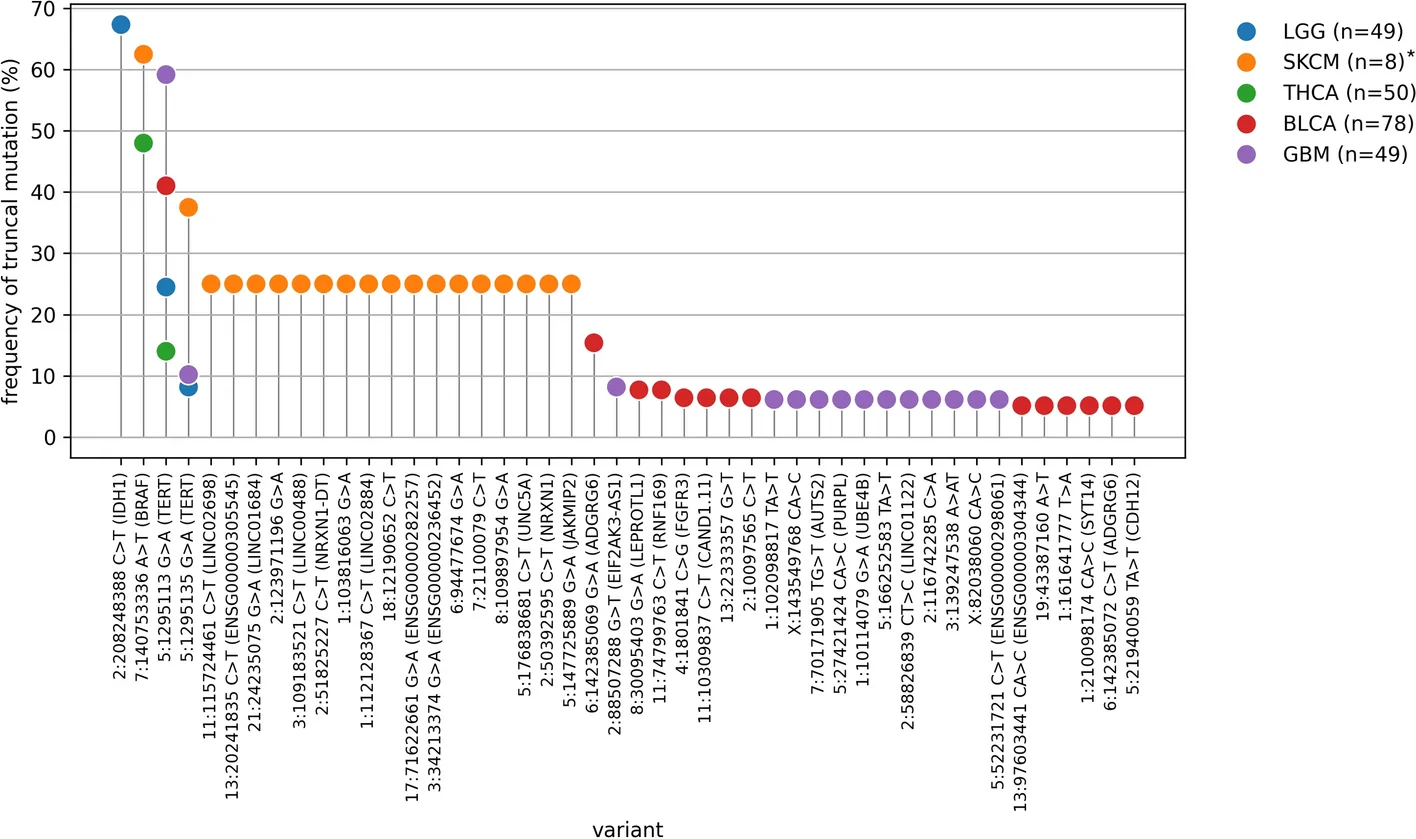
Frequent truncal mutations detected by TruncalFlow across the top five tumor types. The corresponding HUGO gene symbol is shown where available, otherwise Ensemble gene ID or chromosomal position for intergenic variants. *Small sample size; recurrence frequencies should be interpreted with caution

We next assessed the recurrence of truncal mutations (at the nucleotide level) within and across tumor types. Within tumor types, LGG, SKCM, THCA, BLCA, and GBM showed the highest numbers of recurring truncal mutations, with the most frequent event (*IDH1* mutation R132H) detected in 67% of LGG samples (Fig. 3), in line with its known hotspot occurrence in secondary GBM [25].

Several truncal mutations were shared across multiple tumor types, including BRAF V600E and TERT promoter mutations. While our pipeline identified multiple recurrent truncal mutations in the SKCM cohort, the small sample size (n = 8) limits the statistical power of these frequency estimates; therefore, these findings are considered preliminary and descriptive rather than representative of broader SKCM clinical populations.

Some truncal mutations were shared across multiple tumor types, including TERT promoter mutation c.-124C>T in LGG, THCA, BLCA, and GBM, and BRAF V600E present in THCA (48%) and SKCM (62%). While our pipeline also identified other recurrent truncal mutations in the SKCM cohort with frequency above 25%, the small sample size (n = 8) limits the statistical power of these frequency estimates; therefore, the findings regarding SKCM are considered preliminary and descriptive rather than representative of broader SKCM clinical populations.

We further investigated mutation recurrence across tumor types as truncal mutations shared by at least two cancers. The most widespread truncal mutation (5:1295113 G>A (c.-124*C>*T)) was observed in eight tumor types (Fig. S2). COAD, STAD, and READ contributed the highest number of recurrent truncal events across tumor types. Among the top 30 cross-cancer mutations, two TERT promoter variants (5:1295113 G>A (c.-124*C>*T) and 5:1295135 G>A (c.-146C>T)) were detected, consistent with their established role in telomerase activation and tumor progression in glioma, melanoma, thyroid, and bladder cancer [26].

#### Analysis of driver mutations in truncal mutations set

To evaluate the functional relevance of truncal mutations identified by TruncalFlow, variants were annotated against the OncoVar database [27] of curated cancer drivers. For each TCGA tumor type, the corresponding driver list was obtained from OncoVar, and truncal variants were matched using chromosome, position, reference, and alternate alleles. To ensure reliability, only exact matches were retained, and variants with ambiguous alignment or longer (> 2 bp) indels were excluded to reduce potential artifacts.

Out of 17,769 unique truncal mutations detected across all samples, 30 matched entries in OncoVar, corresponding to 13 distinct driver annotations. Although this represents a small fraction of the total, it aligns with the expected rarity of high-confidence driver events. Multiple truncal variants converged on the same driver annotation, most notably eight distinct mutations mapped to *TP53* (Table 2).

*TP53* was the most broadly distributed truncal driver, identified in eight tumor types and serving as the only annotated driver in GBM, HNSC, OV, and STAD (Table 2). *KRAS* was the second most recurrently mutated truncal driver, detected in five tumor types and the sole annotated driver in LUAD, PAAD, and READ. BLCA exhibited the greatest diversity, with five distinct truncal driver mutations (*DMXL2*, *RHOB*, *FGFR3*, *KRAS*, and *PIK3CA*).

Statistical testing confirmed a global enrichment of cancer drivers among truncal mutations when analyzing all tumor types combined (odds ratio = 1.85, *p*-value = *1.07e-14*). At the individual cohort level, this enrichment reached statistical significance (p < 0.05) in four tumor types (Table 3), while the remaining cohorts did not reach significance, likely reflecting limited cohort sizes and the sparsity of annotated driver events in specific datasets (Table S3).

**Table 2:** Annotated truncal driver mutations across tumor types

Driver gene	Tumor types	Number of distinct mutations mapped to the gene
TP53	BRCA, COAD, GBM, HNSC, LGG, OV, STAD, UCEC	8
PIK3CA	BLCA, BRCA, CESC, COAD, SARC, UCEC	6
KRAS	BLCA, COAD, LUAD, PAAD, READ	6
BRAF	SKCM, THCA	1
IDH1	LGG	1
FGFR3	BLCA	1
RHOB	BLCA	1
AKT1	BRCA	1
DMXL2	BLCA	1
FBXW7	UCS	1
HRAS	THCA	1
MAPK1	CESC	1
IDH2	LGG	1

**Table 3:** Summary of truncal vs non-truncal driver and non-driver mutations, shown for tumor types where the enrichment of cancer drivers among truncal mutations reached statistical significance

Tumor type	Truncal drivers	Truncal non-drivers	Non-truncal drivers	Non-truncal non-drivers	Odds ratio	*p*-value	FDR
BRCA	26	172494	2	176697	13.32	1.13e−06	1.13e−05
LGG	51	105884	5	84426	8.13	7.71e−09	1.89e−07
PAAD	29	112717	8	84285	2.71	0.0060	0.0453
THCA	27	34771	4	58985	11.45	1.26e−08	1.89e−07

## Discussion

In this study, we benchmarked five clonal reconstruction tools and introduced TruncalFlow, an open source and scalable pipeline for automated extraction of truncal mutations from tumor sequencing data. Our benchmarking identified QuantumClone with CNV integration as the most balanced approach in terms of sensitivity, specificity, and computational efficiency, and we demonstrated its application across TCGA samples. The pipeline successfully recovered biologically meaningful patterns, including recurrence of well-known driver mutations such as TP53 and KRAS, while also confirming a broader enrichment of truncal variants in known drivers. These findings establish TruncalFlow as a valuable framework for both large-scale cancer genomics research and translational applications.

A key contribution of this work is to facilitate the identification of truncal mutations, which hold particular promise for liquid biopsy and ctDNA–based monitoring. Truncal variants, by definition, are present in every cancer cell, making them more robust markers of overall tumor burden than branch or subclonal mutations. Previous studies have shown that clonality strongly influences ctDNA detectability: founder or truncal mutations typically exhibit higher variant allele frequencies in plasma, whereas branch mutations are often restricted to minor subclones and may go undetected. For example, Abbosh et al. [15] demonstrated that clonal mutations correlate more strongly with ctDNA levels than subclonal ones, and Sasaki et al. reported that founder mutations are significantly more abundant in preoperative ctDNA compared to later-arising variants [28]. By enabling efficient identification of these truncal events from single-sample tumor sequencing, TruncalFlow provides an essential link between tissue-based clonal analysis and liquid biopsy applications.

Our benchmarking choice reflects this clinical priority: while PyClone-VI achieved higher recall with lower variance, its elevated false positive rate (35.89%) risks labeling subtruncal mutations as truncal. In ctDNA monitoring, such errors could track minor subclones that shed unevenly into plasma, yielding misleading tumor burden estimates or false positives during remission surveillance. Conversely, missing truncal mutations (false negatives) reduces the number of available biomarkers but is less clinically damaging than false positives. QuantumClone’s conservative profile ensures high-confidence truncals present in all cells for reliable detection, prioritizing specificity where false truncal calls undermine assay stability more than occasional misses.

Importantly, for monitoring purposes, truncal mutations need not be driver events. While driver mutations like TP53, KRAS, or TERT promoter variants are frequently truncal and biologically relevant, many non-driver variants also arise early and persist in all tumor cells. From the perspective of disease tracking, any truncal mutation, driver or passenger, can serve as a stable molecular barcode of the tumor. In fact, prioritizing only canonical drivers may unnecessarily narrow the set of usable biomarkers. Our results show that the majority of truncal mutations do not overlap with known drivers, yet they remain valid targets for ctDNA assays as long as they reliably reflect tumor burden. This distinction is critical: clinical monitoring does not require functional significance, only sensitivity and stability of detection.

Nevertheless, several limitations should be acknowledged. First, our benchmarking was restricted to single-sample tools, which do not capture the full complexity of spatial or temporal tumor heterogeneity. Expanding TruncalFlow to integrate multi-region and longitudinal sequencing will be essential as such datasets become more accessible. Second, the accuracy of truncal identification is dependent on upstream variant calling and CNV correction; variability among pipelines may affect results. As demonstrated in a large prostate cancer cohort [29], SNV detection tools may substantially alter subclonal diversity and mutation clonality assignments due to differences in low-VAF mutation calling, while CNV approaches primarily influence cellularity estimates. These pipeline differences can elevate false positive rates by misassigning subclonal variants to the truncal cluster or increase false negatives by missing low-prevalence truncal mutations, particularly problematic in noisy real-world sequencing data compared to cleaner simulated DREAM datasets. Lastly, tool performance varies by tumor type, suggesting that no single tool is optimal across all cancer types. This tumor-type dependence may motivate an ensemble approach for truncal mutation identification in practice, where users select tools based on their specific tumor type and whether they prioritize sensitivity or specificity for their use case.

Looking forward, several avenues can enhance the utility of TruncalFlow. One promising direction is to integrate knowledge of well-established mutation patterns in specific cancer types. For instance, TP53 and KRAS are commonly truncal in colorectal and lung cancers, while TERT promoter mutations are often early events in glioma and thyroid cancer. By incorporating such cancer-type–specific expectations, TruncalFlow could prioritize variants that are both clonally stable and likely to be clinically meaningful in that tumor context. This does not replace unbiased detection, but it provides an additional layer of confidence when selecting mutations for translational applications such as ctDNA monitoring.

In summary, TruncalFlow lowers the barrier for extracting truncal mutations from tumor sequencing data and demonstrates the value of focusing on clonality in cancer genomics. By emphasizing that any truncal variant–driver or passenger–can serve as a robust biomarker for ctDNA-based monitoring, this work supports the broader integration of clonal reconstruction into translational oncology. Ultimately, automated workflows such as TruncalFlow will help bridge the gap between complex tumor evolution and practical applications in precision oncology.

## Conclusions

TruncalFlow provides an open-source, scalable solution for automated identification of truncal mutations from tumor sequencing data, facilitating integration of clonal analysis into translational and clinical research. Our benchmarking highlights QuantumClone with CNV integration as the most balanced single-sample tool, and demonstrates that both driver and non-driver truncal variants can serve as stable biomarkers for ctDNA-based monitoring. By enabling efficient, computationally driven extraction of truncal mutations, TruncalFlow bridges the gap between tumor evolutionary modeling and practical applications in cancer detection and surveillance, supporting precision oncology efforts through robust and reproducible clonality assessment.

## Additional file

Supplementary file 1

## Data Availability

For benchmarking, the simulated dataset from the ICGC-TCGA DREAM Somatic Mutation Calling - Tumour Heterogeneity Challenge [18] was used, which contains 51 tumor samples with predefined clonal structures, enabling objective evaluation of computational methods. The dataset was obtained through Synapse Storage [30], using files ’battenberg_files.tar.gz’ and ’truthFiles.tar.gz’. VCF files with SNVs and Battenberg outputs with allele-specific CNVs served as inputs for clonal reconstruction. The ground-truth clonal structures for each sample were used to evaluate the accuracy of each method. For experiments with TruncalFlow, based on QuantumClone, the tool that showed the most suitable trade-off for truncal mutation identification in our benchmarking, we used data from TCGA Research Network [19]. TCGA data were accessed via the Genomic Data Commons (GDC) portal [31], and projects corresponding to 30 tumor types (e.g., ’TCGA-ACC’, ’TCGA-BLCA’, ’TCGA-BRCA’; full list provided in Table S4) were included. For each tumor type, up to 50 tumor samples with matched somatic VCF files and CNV profiles were randomly selected without replacement. For tumor types with fewer than 50 eligible samples, all available samples were included. In total, dataset covers 30 tumor types, with VCF and CNV files analyzed for clonal reconstruction.

## Abbreviations

BLCA: Bladder urothelial carcinoma
BRCA: Breast invasive carcinoma
CNV: Copy number variation
CNS: Copy number alteration
COAD: Colon adenocarcinoma
ctDNA: Circulating tumor DNA
FPR: False positive rate
GBM: Glioblastoma multiforme
HNSC: Head and neck squamous cell carcinoma
indel: Insertion/deletion
KICH: Kidney chromophobe
LGG: Brain lower grade glioma
LIHC: Liver hepatocellular carcinoma
LOH: Loss-of-heterozygosity
LUAD: Lung adenocarcinoma
LUSC: Lung squamous cell carcinoma
MCMC: Markov chain Monte Carlo
MM: Multiple myeloma
OV: Ovarian serous cystadenocarcinoma
PAAD: Pancreatic adenocarcinoma
READ: Rectum adenocarcinoma
SKCM: Skin cutaneous melanoma
SNV: Single nucleotide variant
SSM: Simple somatic mutation
STAD: Stomach adenocarcinoma
TCGA: The cancer genome atlas
THCA: Thyroid carcinoma
TPR: True positive rate
VAF: Variant allele frequency
VCF: Variant calling format

## Availability and requirements

Project name: TruncalFlow Project home page: https://github.com/OncoRNALab/TruncalFlow Operating system(s): Platform independent Programming languages: Python, R Other requirements: PBS scheduler (for ’*–executor pbs*’); Singularity/Apptainer; dependencies via *singularity pull docker://stevelefever/thesis_2025_marina*. Recommended: 1≤ CPUs, 4–32 GB RAM scaling with number of variants and/or samples. License: MIT License Any restrictions to use by non-academics: no restrictions.
