# Supplementary material for: Integrative benchmarking and automation of clonal reconstruction of somatic mutations in single-sample tumor genome analysis
Source: BMC Bioinformatics. 2026 Aug 5;27:256. doi: 10.1186/s12859-026-06589-8 (PMC13726195; doi:10.1186/s12859-026-06589-8)
Supplement: Supplementary file 1 [file 12859_2026_6589_MOESM1_ESM.pdf]

Table S1 Summary of clonal reconstruction methods' performance metrics.

Method	Weighted average TPR	Weighted average FPR	Runtime	Memory used
PhyloWGS	91.49%	12.75%	6 h – 5 days	High-memory nodes
QuantumClone	63.42%	6.85%	10 min – 10 h	30 Gb/sample, 8 CPUs
QuantumClone + CNV	88.75%	12.43%	10 min – 12 h	30 Gb/sample, 8 CPUs
PyClone-VI	95.28%	35.89%	1 h – 5 h	20 Gb/sample, 8 CPUs
FastClone	87.72%	25.16%	10 s – 4 min	20 Gb/sample, 8 CPUs

TPR, true positive rate; FPR, false positive rate. Runtime and memory estimates are approximate ranges for benchmark datasets.

Table S2 Chi-squared test results of indel enrichment in truncal mutations for COAD and READ.

Tumor Type	Chi-squared	p-value
COAD	5367.14	$p < 1 \times 10^{-10}$
READ	3839.72	$p < 1 \times 10^{-10}$

Table S3 Summary of truncal vs non-truncal driver and non-driver mutations across all tumor types in the study.

Tumor	Trunc. drv	Trunc. non-drv	Non-trunc. drv	Non-trunc. non-drv	Odds ratio	p-value	FDR
ACC	0	34732	0	33256	–	1.0	1.0
BLCA	52	643654	55	525085	0.7713	0.9252	1.0
BRCA	26	172494	2	176697	13.3168	1.13e-06	1.13e-05
CESC	18	325697	3	187965	3.4627	0.0237	0.0899
CHOL	12	31141	2	12562	2.4203	0.1867	0.4309
COAD	41	350591	15	227922	1.7770	0.0341	0.1022
DLBC	0	105827	0	79258	–	1.0	1.0
ESCA	3	155525	2	100645	0.9707	0.6948	0.9474
GBM	16	265610	4	108829	1.6389	0.2665	0.4996
HNSC	30	307232	20	289299	1.4124	0.1443	0.3609
KICH	0	25411	0	30211	–	1.0	1.0
KIRC	1	171767	2	90972	0.2648	0.9585	1.0
KIRP	29	225087	2	70452	4.5385	0.0121	0.0728
LGG	51	105884	5	84426	8.1329	7.71e-09	1.89e-07
LUAD	28	261685	22	241212	1.1732	0.3382	0.5340
LUSC	21	183466	17	289165	1.9470	0.0292	0.0973
MESO	0	28863	0	10486	–	1.0	1.0
OV	13	254251	4	171003	2.1859	0.1224	0.3338
PAAD	29	112717	8	84285	2.7106	0.0060	0.0453
PCPG	1	16339	0	27505	–	0.3727	0.5590
PRAD	4	82932	5	78537	0.7576	0.7721	1.0
READ	17	217661	7	124410	1.3881	0.3067	0.5340
SARC	6	140194	0	120881	–	0.0240	0.0899
SKCM	8	68035	1	18056	2.1231	0.4074	0.5820
STAD	21	225456	14	204052	1.3576	0.2364	0.4996
TGCT	2	19955	1	31776	3.1848	0.3316	0.5340
THCA	27	34771	4	58985	11.4506	1.26e-08	1.89e-07
THYM	0	17691	0	20971	–	1.0	1.0
UCEC	43	188035	7	73137	2.3893	0.0158	0.0789
UCS	4	62788	0	25409	–	0.2569	0.4996

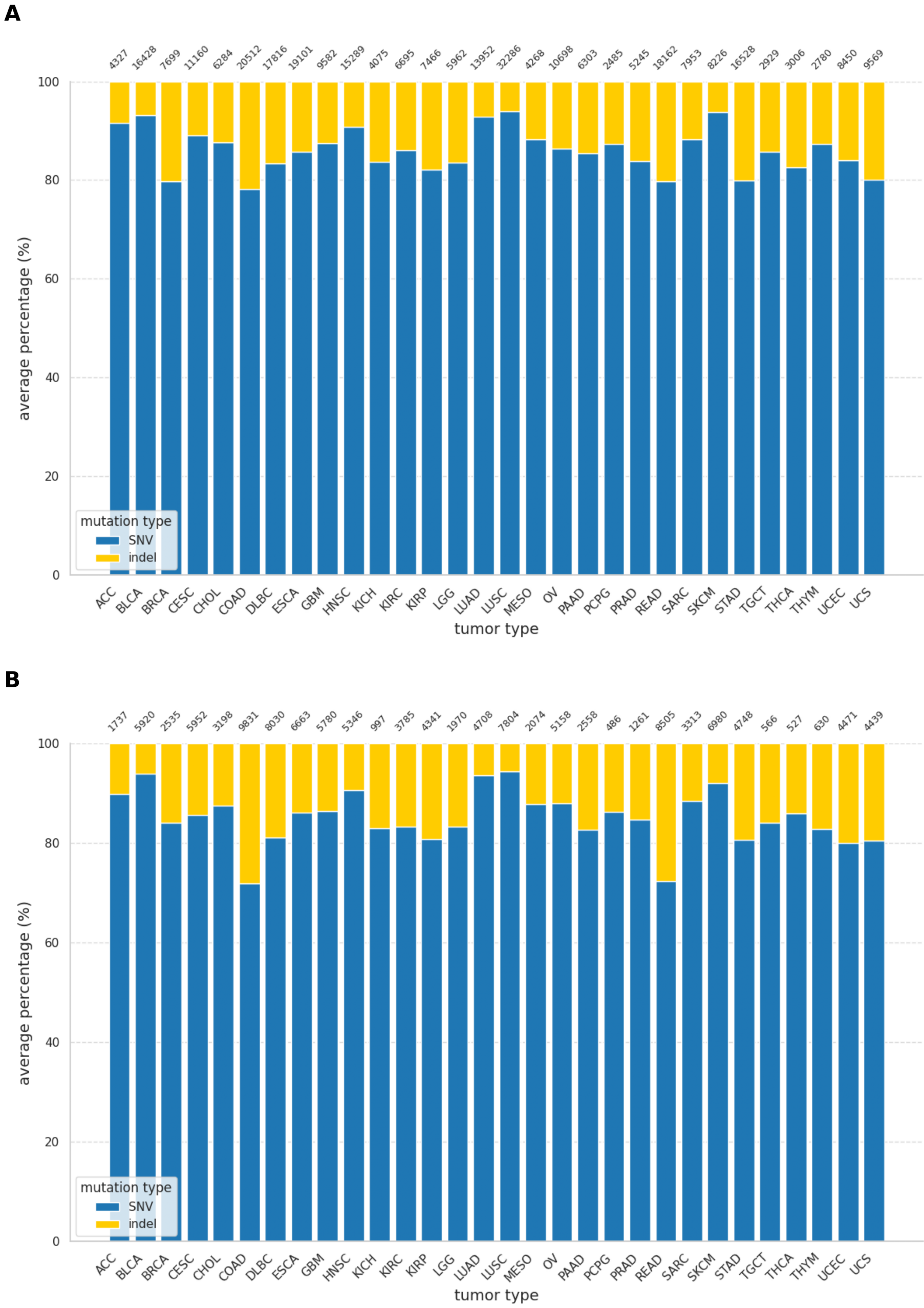

Fig. S1 Distribution of mutation types across 30 TCGA tumor types.
A Average percentage of SNVs and indels per tumor in the original VCF data, grouped by tumor type.
B Average percentage of SNVs and indels per tumor among truncal mutations identified by TruncalFlow.

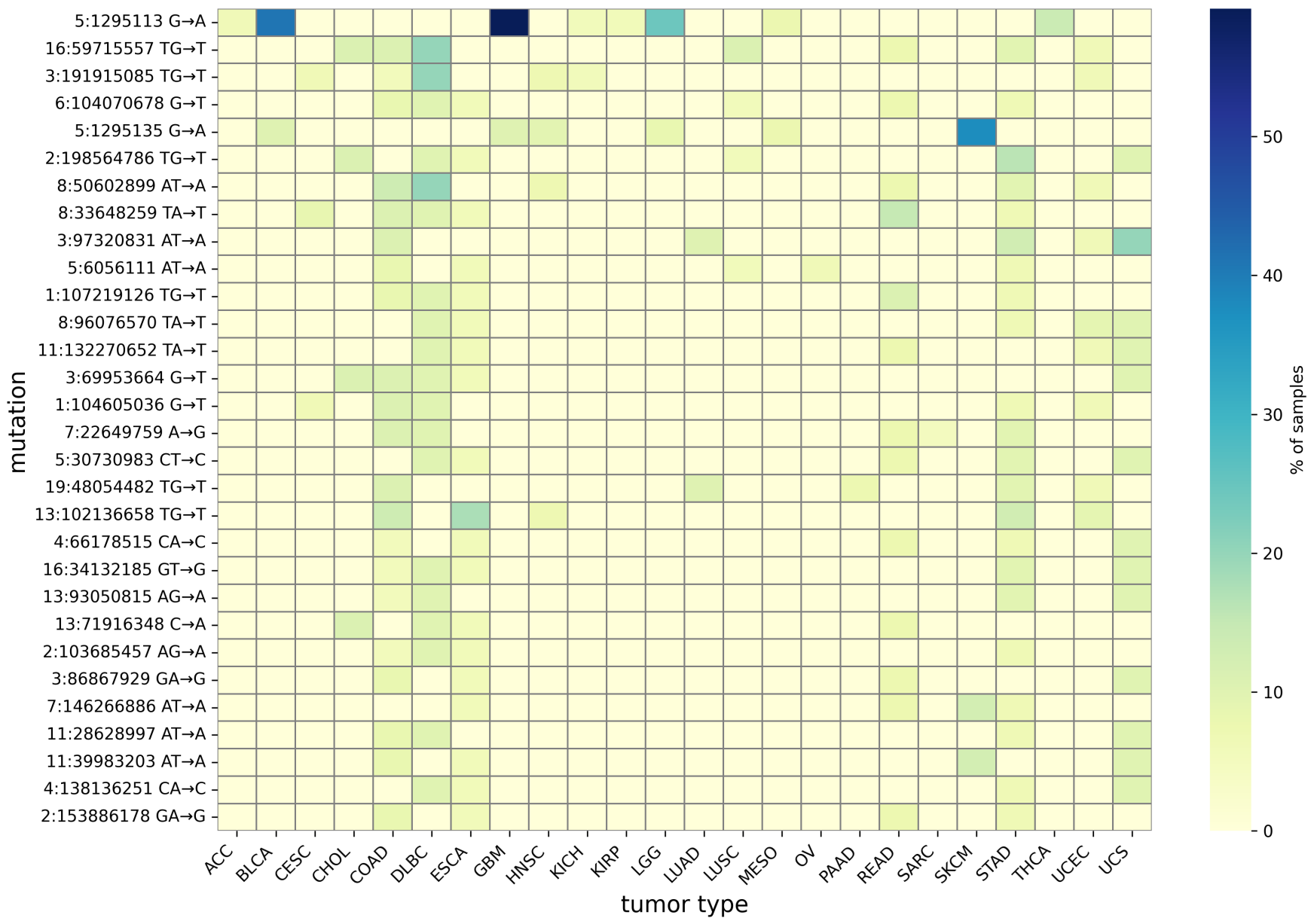

Fig. S2 Recurrently identified truncal mutations across multiple tumor types. Heatmap of the top 30 truncal mutations, ranked by the number of distinct tumor types in which they were detected. The color scale represents the percentage of samples within each tumor type that harbor the mutation, relative to the total number of samples in that tumor type.

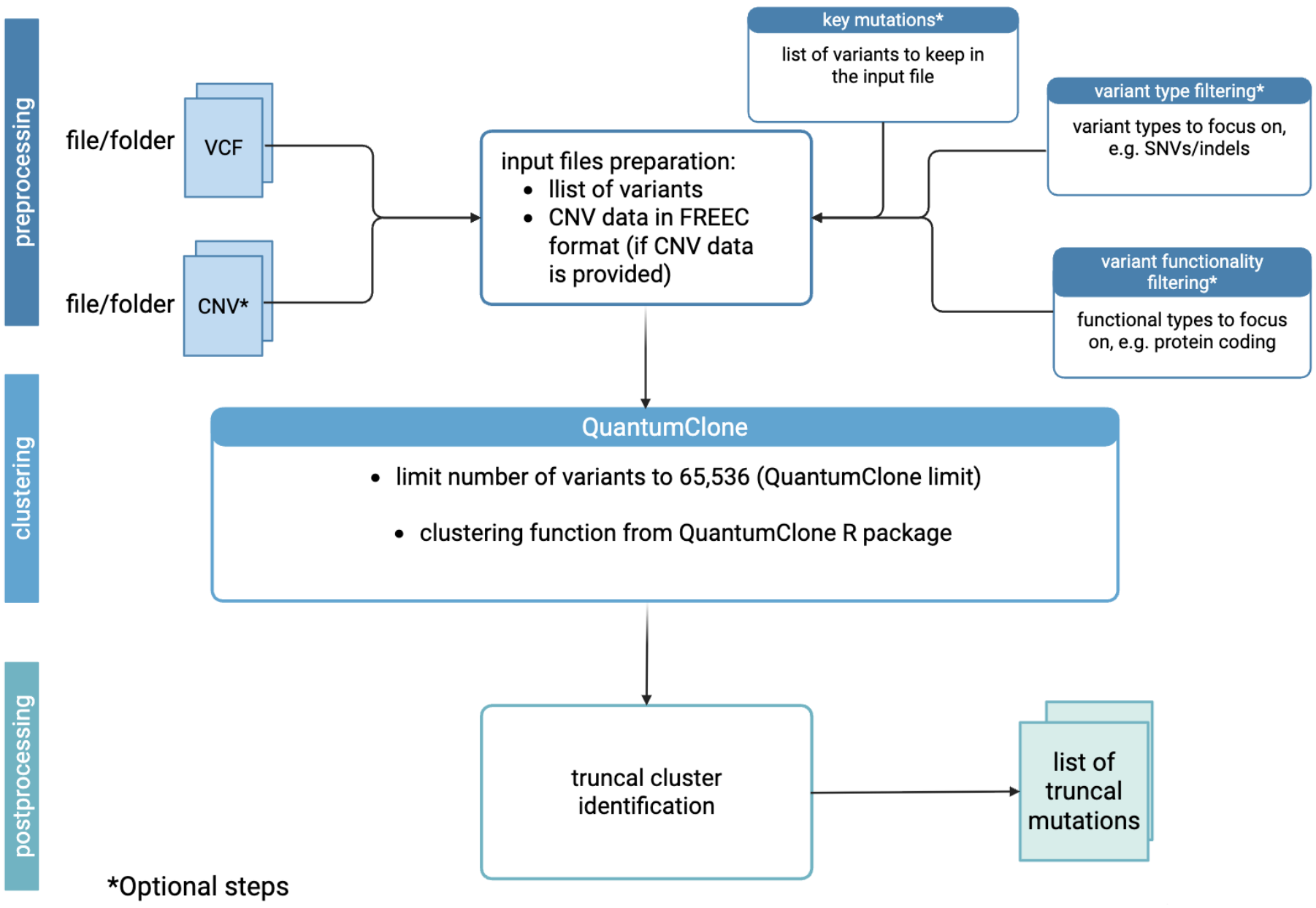

Fig. S3 Overview of TruncalFlow.
The workflow begins with the preprocessing step, which includes formatting of the input data and optional filtering of variants, specified by the user in the command line. Next, it proceeds with the clustering, which is the main step of the pipeline and is based on the QuantumClone algorithm. TruncalFlow completes the job by postprocessing, extracting truncal mutations, and generating a clear output in the format of a truncal mutations list.

Table S4 TCGA project identifiers corresponding to tumor types analyzed in this study.

Tumor type	TCGA project ID
ACC	TCGA-ACC
BLCA	TCGA-BLCA
BRCA	TCGA-BRCA
CESC	TCGA-CESC
CHOL	TCGA-CHOL
COAD	TCGA-COAD
DLBC	TCGA-DLBC
ESCA	TCGA-ESCA
GBM	TCGA-GBM
HNSC	TCGA-HNSC
KICH	TCGA-KICH
KIRC	TCGA-KIRC
KIRP	TCGA-KIRP
LGG	TCGA-LGG
LUAD	TCGA-LUAD
LUSC	TCGA-LUSC
MESO	TCGA-MESO
OV	TCGA-OV
PAAD	TCGA-PAAD
PCPG	TCGA-PCPG
PRAD	TCGA-PRAD
READ	TCGA-READ
SARC	TCGA-SARC
SKCM	TCGA-SKCM
STAD	TCGA-STAD
TGCT	TCGA-TGCT
THCA	TCGA-THCA
THYM	TCGA-THYM
UCEC	TCGA-UCEC
UCS	TCGA-UCS
